# Clinically Robust Deep Learning for Contrast-Enhanced Mammography: Multicenter Evaluation Across Convolutional Neural Network Architectures

**DOI:** 10.3390/bioengineering13040475

**Published:** 2026-04-17

**Authors:** Roberta Fusco, Vincenza Granata, Paolo Vallone, Teresa Petrosino, Maria Daniela Iasevoli, Roberta Galdiero, Mauro Mattace Raso, Davide Pupo, Filippo Tovecci, Annamaria Porto, Gerardo Ferrara, Modesta Longobucco, Giulia Capuano, Roberto Morcavallo, Caterina Todisco, Fabiana Antenucci, Mario Sansone, Mimma Castaldo, Daniele La Forgia, Antonella Petrillo

**Affiliations:** 1 Radiology Division, Istituto Nazionale Tumori-IRCCS-Fondazione G. Pascale, 80131 Naples, Italy; r.fusco@istitutotumori.na.it (R.F.); p.vallone@istitutotumori.na.it (P.V.); t.petrosino@istitutotumori.na.it (T.P.); m.iasevoli@istitutotumori.na.it (M.D.I.); r.galdiero@istitutotumori.na.it (R.G.); m.mattaceraso@istitutotumori.na.it (M.M.R.); davide.pupo@istitutotumori.na.it (D.P.); f.tovecci@istitutotumori.na.it (F.T.); annamaria.porto@istitutotumori.na.it (A.P.); a.petrillo@istitutotumori.na.it (A.P.); 2 Pathology Division, Istituto Nazionale Tumori-IRCCS-Fondazione G. Pascale, 80131 Naples, Italy; gerardo.ferrara@istitutotumori.na.it; 3 Struttura Semplice Dipartimentale di Radiodiagnostica Senologica—IRCCS Istituto Tumori Giovanni Paolo II, 70124 Bari, Italy; m.longobucco@oncologico.bari.it (M.L.); g.capuano@oncologico.bari.it (G.C.); r.morcavallo@oncologico.bari.it (R.M.); todisco.katia@gmail.com (C.T.); d.laforgia@oncologico.bari.it (D.L.F.); 4 Biomedical Engineering Faculty, Università Degli Studi di Napoli Federico II, 80125 Naples, Italy; f.antenucci@studenti.unina.it (F.A.); msansone@unina.it (M.S.); 5 Unit of “Progettazione e Manutenzione Edile ed impianti”, Istituto Nazionale Tumori IRCCS Fondazione Pascale, IRCCS di Napoli, 80131 Naples, Italy; m.castaldo@istitutotumori.na.it

**Keywords:** contrast-enhanced mammography, breast cancer, deep learning, convolutional neural networks, artificial intelligence, lesion classification

## Abstract

Background: This study investigates the impact of anatomically constrained preprocessing and deep learning architecture selection on benign versus malignant breast lesion classification in contrast-enhanced mammography (CEM), with the goal of improving robustness and clinical reliability across heterogeneous data sources. Methods: In this retrospective multicenter study, CEM images from 300 patients (314 lesions) were combined with 1003 publicly available CEM images, yielding a total of 1120 breast cases. Automatic breast segmentation was performed using the LIBRA framework to generate breast-mask images. Eleven deep learning models, including classical convolutional neural networks, attention-based networks, hybrid convolutional neural networks (CNNs), Transformer architectures, and mammography-specific models, were trained and evaluated using both original DICOM images and breast-mask inputs. Performance was assessed using accuracy, balanced accuracy, sensitivity, specificity, AUROC, and AUPRC on cross-validation and independent test sets. Hyperparameter optimization was conducted for the best-performing architecture. Results: Models trained on breast-mask images consistently outperformed those trained on original DICOM images across all architectures and metrics, with AUROC improvements ranging from +0.06 to +0.21. Among all models, ResNet50 trained on breast-mask images achieved the best performance (AUROC = 0.931; AUPRC = 0.933; balanced accuracy = 0.834), further improved after optimization (balanced accuracy = 0.886; sensitivity = 0.842; specificity = 0.930). Classical CNN architectures demonstrated performance comparable to or exceeding that of more complex hybrid CNN–Transformer models when anatomically focused preprocessing and rigorous optimization were applied. Conclusions: Anatomically constrained preprocessing through breast-mask segmentation substantially enhances deep learning performance and stability in CEM-based breast lesion classification. These findings indicate that input representation quality and training optimization are critical determinants of clinically relevant performance, often outweighing architectural complexity, and may support more reliable AI-assisted decision support in CEM workflows.

## 1. Introduction

Breast cancer remains the most frequently diagnosed malignancy in women worldwide, and early detection continues to represent a decisive factor in improving prognosis and survival outcomes [1,2]. Although screening mammography has significantly reduced mortality, its diagnostic performance is limited in women with dense breast tissue, where lesion conspicuity decreases due to reduced contrast between tumoral and fibroglandular structures [1,2,3,4]. To overcome these limitations, functional imaging techniques such as contrast-enhanced mammography (CEM) and dynamic contrast-enhanced magnetic resonance imaging (DCE-MRI) have progressively gained clinical relevance [3,4,5,6,7,8].

CEM combines high-resolution digital mammography with iodinated contrast administration, enabling visualization of tumor-related neoangiogenesis through dual-energy subtraction imaging [5,6,7,8,9,10,11,12,13,14]. This hybrid morphological–functional approach improves sensitivity, particularly in dense breasts and diagnostic problem-solving settings [5,6,7,8,9,10,11,12,13,14]. While DCE-MRI remains the most sensitive modality for breast cancer detection and characterization [4,8], it is associated with higher costs, longer acquisition times, and limited accessibility. Consequently, CEM has emerged as a cost-effective and widely deployable alternative that preserves functional information while maintaining compatibility with mammographic workflows.

Despite these advances, interpretation of CEM remains operator-dependent and may be influenced by subtle enhancement patterns and background parenchymal variability. In this context, radiomics and artificial intelligence (AI) have introduced quantitative frameworks capable of extracting imaging biomarkers beyond human visual perception [6,11,15,16,17,18,19,20,21,22,23,24,25,26,27,28,29,30]. Multiple studies have demonstrated the potential of machine learning and deep learning approaches for lesion characterization, receptor status prediction, and molecular subtype inference using MRI and CEM data [11,15,16,17,18,19,20,21,22,23,24,25,26,27,28,29,30], highlighting the growing role of computational imaging in precision oncology.

In this context, our group has previously investigated radiomics-based machine and deep learning approaches applied to combined CEM and DCE-MRI datasets, demonstrating that handcrafted radiomic features, particularly wavelet-derived texture descriptors extracted from both mammographic projections and MRI enhancement phases, achieve excellent discriminative performance for benign versus malignant lesion classification (Gradient Boosting Machine, AUC = 0.907) and provide informative, albeit more moderate, predictions of histological grade and HER2 receptor status [30]. While those results established the diagnostic value of engineered radiomic signatures from multiparametric imaging, several important limitations remained: the analysis was restricted to a relatively small cohort (n = 153), relied exclusively on handcrafted feature engineering rather than end-to-end representation learning, and did not systematically explore the impact of input representation or architectural design on model behavior. Subsequently, a focused engineering analysis using two standard convolutional architectures—VGG16 and MobileNetV2—on the publicly available CDD-CESM dataset provided initial controlled evidence that LIBRA-based anatomical breast masking systematically improves discrimination performance, sensitivity to malignant cases, and training stability compared to raw DICOM inputs [31]. That study confirmed anatomical constraint as a first-class design variable in CEM-based deep learning pipelines. However, it was confined to two architectures and a single public dataset, leaving open the question of whether such improvements generalize across broader and more heterogeneous architectural families, including hybrid convolutional neural network (CNN) –Transformer and mammography-specific models, and whether they persist in multicenter settings that combine institutional and public data. The present study addresses both remaining gaps: by extending the architectural evaluation to eleven models and incorporating a larger multicenter dataset (n = 1120), it provides a comprehensive and externally grounded assessment of the interplay between preprocessing, architecture, and clinical generalizability in CEM-based deep learning.

Importantly, the scientific contribution of the present work is not limited to confirming the utility of breast-mask preprocessing across a broader set of architectures. Rather, it establishes a novel and clinically relevant finding: that anatomically constrained preprocessing, when combined with rigorous training optimization, renders classical CNN architectures competitive with and in several cases superior to more complex hybrid CNN–Transformer and mammography-specific models. This result challenges the prevailing assumption in the field that architectural sophistication is the primary determinant of deep learning performance in breast imaging, and instead positions input representation quality and training strategy as first-class design variables. Furthermore, the multicenter experimental design, integrating institutional data from two IRCCS centers with the publicly available CDD-CESM archive, provides an externally grounded validation framework that goes substantially beyond the scope of any single-dataset, two-architecture study. We acknowledge the reviewer’s observation that the eleven models are established architectures and that LIBRA preprocessing benefits were initially documented in [31]. Scientific contribution in applied engineering research is not limited to algorithmic invention: the present study provides the first multicenter evidence that the preprocessing performance benefit generalizes uniformly across five distinct architectural families and heterogeneous institutional datasets (n = 1120). This reproducible engineering finding provides evidence-based design guidance for CEM AI systems, demonstrating that input representation engineering yields consistent performance gains independently of architectural choice. Early CNN architectures such as VGG [31] and ResNet [32] demonstrated promising results in mammographic abnormality detection. More recently, domain-adapted and hybrid architectures—including GLAM-Net, ViT-Mammo, FCCS-Net, and Transformer-based frameworks—have been proposed to better capture multi-view correlations and global contextual information in breast imaging [33,34,35,36,37,38,39,40,41,42,43,44,45,46]. However, while architectural innovation has been extensively explored, comparatively less attention has been devoted to systematic multicenter validation and to the role of input representation in shaping model robustness.

The present work addresses a systematic optimization problem evaluating how preprocessing pipeline design architectural selection and hyperparameter optimization determine AI performance in CEM-based breast lesion classification. The LIBRA breast-mask preprocessing is itself an engineering contribution, applying image-processing operations from biomedical signal theory to constrain the input representation with quantifiable and reproducible effects on model performance. The finding that input representation engineering can substitute for architectural complexity provides direct design guidance for clinical AI systems. From a biomedical engineering perspective, image preprocessing and anatomical constraint may substantially influence learning behavior. Variability related to acquisition protocol, device characteristics, anatomical differences, and contrast kinetics can introduce non-diagnostic signals that deep learning systems may inadvertently exploit [34,35,47,48,49,50]. In mammography and CEM, background structures such as pectoral muscle, air regions, and acquisition artifacts may confound feature learning if not properly controlled for. Prior studies suggest that intensity normalization, histogram-based enhancement, and segmentation-guided analysis can improve lesion conspicuity and downstream model stability [35,36,37,47,48,49,50]. Nevertheless, the relative contribution of input representation compared with architectural complexity remains insufficiently characterized in CEM.

In this study, we perform a comprehensive multicenter evaluation of deep learning models for benign versus malignant lesion classification in contrast-enhanced mammography. Eleven architectures, including classical CNNs, attention-augmented networks, hybrid CNN, Transformer models, and mammography-specific systems, are evaluated under identical training conditions using both original DICOM inputs and anatomically constrained breast-mask representations. We assess discrimination performance, sensitivity–specificity balance, and generalization across heterogeneous data sources. By systematically comparing architectures and input representations, we aim to determine whether clinically robust performance in CEM. Building on our prior radiomic analysis [30] and the initial controlled deep learning investigation [31], both of which established the value of CEM-derived imaging biomarkers and anatomical preprocessing, this study represents a systematic and comprehensive progression toward a multicenter, multi-architecture deep learning framework, addressing scalability, architectural diversity, and generalizability across heterogeneous data sources.

## 2. Methods

### 2.1. Patient Selection

Patient enrollment took place between October 2017 and January 2025. The retrospective, multicenter study was conducted following approval from the local Institutional Review Board (Protocol No. 67/25 OSS of National Cancer Institute IRCCS of Naples Pascale Foundation). Participants provided written informed consent. A total of 300 patients with 314 breast lesions were included in the analysis, each having undergone CEM imaging. The cohort had a mean age of 52.8 years (±12.3), with ages ranging from 25 to 92 years. The inclusion criteria comprised patients with histologically confirmed breast lesions who underwent CEM imaging as part of their preoperative staging. This study involved two institutions: the Istituto Nazionale Tumori–IRCCS–Fondazione G. Pascale (Naples) and the Oncological Institute of Bari (Bari). Exclusion criteria included the presence of breast implants, pregnancy or suspected pregnancy, inability to remain still during imaging, renal impairment, or ongoing chemotherapy at the time of examination.

### 2.2. Imaging Protocol

The same acquisition protocol was implemented by centers. The CEM protocol included image acquisition in both cranio-caudal (CC) and mediolateral oblique (MLO) projections, initiated two minutes following intravenous injection of an iodinated contrast agent (Visipaque 320; GE Healthcare, Inc., Princeton, NJ, USA) at a dosage of 1.5 mL/kg body weight and an injection rate of 2–3 mL/s. Additional image series were acquired at approximately four and eight minutes post-injection, again in both CC and MLO views. Each CEM exam comprised a dual-energy acquisition: a low-energy (LE) exposure (26–30 kVp) and a high-energy (HE) exposure (45–49 kVp). The resulting LE and HE images were digitally subtracted to generate recombined images that emphasize areas of contrast uptake, providing functional insight into lesion vascularization. Post-processing of all CEM images was centralized and conducted at a single institution, encompassing both segmentation and CNN model analysis.

#### CDD-CESM Archive from Cancer Imaging Archive

To increase image number for CNN training, a public dataset was considered: the CDD-CESM Archive from the Cancer imaging archive [34]. This dataset is a collection of 1003 high-resolution contrast-enhanced spectral mammography (CESM) images with annotations and medical reports. CESM is done using standard digital mammography equipment, with additional software that performs dual-energy image acquisition. Two minutes after intravenously injecting the patient with non-ionic low-osmolar iodinated contrast material (dose: 1.5 mL/kg), cranio-caudal (CC) and mediolateral oblique (MLO) views are obtained. Each view comprises two exposures, one with low energy (peak kilo-voltage values ranging from 26 to 31 kVp) and one with high energy (45 to 49 kVp). Low- and high-energy images are then recombined and subtracted through appropriate image processing to suppress the background breast parenchyma. A complete examination is carried out in about 5–6 min. For our analysis, the 1003 CEM subtracted images of the CDD-CESM Archive were used.

### 2.3. Histopathological Analysis

Histopathological evaluation of tissue specimens was considered the diagnostic gold standard. Both tumor grade and HER2 status were assessed through immuno-histochemical analysis. Tumor grading was performed according to the modified Scarff–Bloom–Richardson system, as refined by Elston and Ellis, which classifies tumors into three grades based on tubule formation, nuclear pleomorphism, and mitotic count. HER2 (human epidermal growth factor receptor 2) expression was evaluated using IHC staining, with scores ranging from 0 to 3+. Tumors were classified as HER2-positive when strong complete membrane staining was observed in more than 10% of tumor cells (score 3+). In cases with an equivocal IHC result (score 2+), HER2 amplification was confirmed using fluorescence in situ hybridization (FISH), in accordance with ASCO/CAP guidelines. The identification of HER2 overexpression is clinically relevant, as it is associated with a more aggressive tumor phenotype and potential eligibility for targeted therapies such as trastuzumab.

### 2.4. Segmentation of the Breast Region

Before image processing, we used the LIBRA system (BreastCancer LIBRA, released as part of CaPTk [7,8]) for automatic segmentation of the breast region in the original CEM DICOM images. LIBRA is a dedicated algorithm for mammographic breast region extraction that employs intensity-based filtering, morphological refinement, and anatomical priors to separate the breast tissue from the background, pectoral muscle, and acquisition artifacts.

This tool has been widely used in the literature for quantitative breast imaging analysis and provides robust and reproducible breast masks.

In our workflow, LIBRA was applied directly to the raw CEM DICOM images to generate a binary breast mask for each view. These masks were then used to derive an alternative set of inputs, referred to in this work as LIBRA breast masks [7,8]. Then, the performance of all deep learning models was evaluated using two complementary input modalities: (i) the original enhanced images, and (ii) the corresponding breast masks produced by LIBRA. This dual evaluation allowed us to assess the contribution of breast morphology alone to compare model performance across anatomically constrained versus full-image inputs.

### 2.5. Image-Processing Pipeline

In this work, a mammography image classification model is proposed, encompassing three main processing stages: image normalization and enhancement, breast region segmentation, and deep-learning-based classification. Eleven CNN architectures were tested [31,32,33,35,36,37,38,39,40,41,42,43,44,45,46]. The working of each block is explained in the following subsections.

#### 2.5.1. Image Enhancement and Segmentation

The enhancement block is designed to standardize mammography inputs and improve the visibility of diagnostically relevant structures prior to learning. As a preliminary normalization step, each DICOM mammogram undergoes percentile-based intensity windowing (5th–99.5th percentile), which acts as a contrast-stretching mechanism to suppress outliers and harmonize acquisition variability. Pixel intensities are subsequently normalized to the [0, 1] range and converted into an 8-bit representation, after which images are resized to a fixed spatial resolution to ensure input homogeneity [35].

Following normalization, a Global Histogram Matching (GHM) procedure is applied to reduce inter-patient and inter-device variability. A global reference cumulative distribution function (CDF) is computed over the entire dataset, excluding background regions, and each image histogram is transformed to match this reference distribution. This type of histogram-based contrast normalization has been widely adopted in mammographic enhancement pipelines and is reported to improve lesion conspicuity and downstream learning stability [47].

To enhance local structures, we apply a Local Contrast Mapping (LCM) operator, which sharpens tissue boundaries by subtracting a Gaussian-smoothed version of the image, similar to the LCM-based local enhancement strategy described by Vaishya et al. [36] for mammographic lesion detection. This is followed by Contrast-Limited Adaptive Histogram Equalization (CLAHE), which improves micro-contrast without excessively amplifying noise—a well-established technique also adopted in previous mammographic processing pipelines to enhance faint calcification and soft-tissue structures [36].

Finally, a denoising stage is applied, implemented via wavelet-based filtering or non-local means, depending on library availability. Such filtering strategies have been shown to improve Peak Signal-to-Noise Ratio (PSNR) and lesion visibility, which is particularly beneficial in the context of enhancing fine structures such as microcalcifications. Overall, this enhancement pipeline produces highly standardized, contrast-balanced, and noise-suppressed images that are optimally prepared for segmentation and learning.

All preprocessing operations (percentile-based windowing, intensity normalization, Global Histogram Matching, CLAHE, and denoising) were applied uniformly to the entire dataset before data splitting. These transformations are purely image-based, do not incorporate class labels, and do not involve model-driven parameter estimation. For this reason, they do not introduce data leakage. Their purpose is to harmonize acquisition variability across institutions and devices, ensuring that all images share a consistent intensity distribution prior to training. Since no statistics are computed in a way that depends on the training/test partition or on label information, preprocessing acts as a global, deterministic standardization step and does not bias the evaluation process.

After enhancement, a global Otsu thresholding algorithm is applied. Otsu’s method, a non-parametric, unsupervised global thresholding strategy, remains a widely used approach in mammographic segmentation due to its robustness and simplicity, as also reported in Vaishya et al.’s work [36], where it was effectively combined with morphological refinement to extract abnormal regions in mammograms.

Since Otsu’s threshold may inconsistently select either the dense or fatty component depending on the histogram pattern, we automatically evaluate both binary configurations and select the one best corresponding to plausible breast anatomy based on area coherence criteria. The selected binary mask is refined through morphological closing to fill holes and connect fragmented components, followed by opening to remove small isolated artifacts [34,36].

#### 2.5.2. Learning and Classification

The learning block is responsible for training a deep neural classifier on mammograms to distinguish malignant from benign/normal cases. Our framework supports eleven state-of-the-art architectures, including ResNet, DenseNet, EfficientNet, MobileNetV2, hybrid CNN-Transformer networks, and domain-specific mammography models such as GLAM-Net and TransBreastNet (Table 1). The value of hybrid CNN-Transformer architectures for breast imaging has been recently demonstrated extensively in the literature: for example, TransBreastNet integrates convolutional spatial encoding with Transformer-based temporal modeling to improve multi-task mammographic diagnosis [37], while GLAM-Net and ViT-Mammo incorporate attention mechanisms tailored to breast anatomy and achieve superior diagnostic accuracy compared to general-purpose backbones (Table 1) [34].

Training is performed using a supervised learning paradigm with balanced sampling and cross-entropy loss. The classifier head is trained first, followed by fine-tuning of the backbone with a differential learning rate strategy, where the classifier uses a higher learning rate than the feature extractor. Optimization employs the Adam optimizer with cosine annealing scheduling and optional gradient clipping for stability.

To mitigate class imbalance, we employ weighted loss functions and stratified folds, consistent with recent best practices in large-scale mammography benchmarks, such as those reported by Sharma et al. [34]. Multi-view fusion is performed by averaging posterior probabilities across available mammographic projections, aligning with evidence from multi-view architectures such as GLAM-Net, which demonstrate superior performance when integrating CC and MLO views.

We calculated the Balanced Accuracy (BalAcc), defined as the average of sensitivity and specificity, which provides a more robust assessment under class imbalance and better reflects clinical utility.

Sensitivity (recall for malignant cases) and specificity (true-negative rate) were included to quantify the model’s ability to correctly identify malignant lesions while avoiding clinically costly false positives.

We also evaluated performance using the Area Under the Receiver Operating Characteristic Curve (AUROC) and the Area Under the Precision–Recall Curve (AUPRC).

All metrics were computed on each cross-validation fold and on the independent held-out test set. The results are reported as point estimates derived from the best-performing model configurations identified during training. For test-set proportional metrics (sensitivity and specificity), 95% confidence intervals (CIs) were estimated using the Wilson score method. For AUROC, 95% CIs were estimated using the DeLong method.

Decision thresholds (Thr) for malignancy detection were consistently selected using the Youden index as the primary operating criterion throughout this study. The Youden index provides a balanced trade-off between sensitivity and specificity.

In scenarios characterized by a pronounced imbalance between sensitivity and specificity, additional analyses were conducted to assess whether calibration of the decision threshold could mitigate this effect. In particular, probability calibration techniques were investigated on validation data to evaluate their impact on the operating point defined by the Youden index. These analyses were performed exclusively on validation prediction, and the resulting thresholds were subsequently applied unchanged to the independent test set.

Final performance evaluation was conducted on a held-out test set.

#### 2.5.3. Configuration Settings

All experiments were conducted using a fixed configuration. Training was performed using a batch size of 32, a maximum of 30 epochs per fold, and a 5-fold stratified cross-validation scheme. To control class imbalance at the case level, the training set was always balanced using the moderate undersampling strategy implemented in the code with a moderate undersampling ratio fixed to 2.0. This approach ensured that the majority class was reduced in a controlled manner without artificially replicating minority cases. A balanced train–test split was generated by assigning 15% of the malignant cases to the test set and matching them with an equal number of benign/negative cases. This partitioning was performed prior to any cross-validation procedure, and the test set was kept completely uninvolved in model training, fold assignment, model selection, and hyperparameter tuning throughout the entire experimental workflow, ensuring full independence of the final evaluation.

After identifying the best models, we performed an additional hyperparameter optimization stage to further refine the best-performing architectures. To this end, we implemented a structured grid search procedure aimed at identifying the optimal combination of learning parameters, including learning rate, backbone learning rate, dropout rate, undersample ratio, and augmentation. The batch size was kept fixed at 32 for all configurations.

Specifically, the grid search varied the classifier head learning rate (H LR: 1 × 10^−3^, 1 × 10^−4^), the backbone learning rate (B LR: 1 ×10^−4^, 3 × 10^−5^), the dropout rate in the final classification layer (Drop: 0.2, 0.3, 0.5), the majority-class undersampling ratio at the case level (R: 1.5, 2.0), and the strength of data augmentation (Aug: light augmentation versus stronger augmentation). The light augmentation strategy consists of minimal geometric perturbations designed to preserve breast anatomy while introducing mild variability. This setting included random horizontal flipping (*p* = 0.5), rotations up to 10°, and affine transformations with translation (±5%), scale factor (0.9–1.1), and shear (±8°). A strong augmentation strategy consists of a broader and more challenging set of perturbations, including random horizontal flipping (*p* = 0.5), rotations up to 15°, and affine transformations with translation (±10%), scale factor (0.85–1.15), and shear (±12°).

For each combination in the grid, a model instance was trained using a 3-fold stratified cross-validation scheme on the training + validation split. To reduce computational cost while preserving comparability, each fold was trained for a maximum of 5 epochs (including an initial 2-epoch warm-up phase during which the backbone remained frozen), with cosine-annealing learning-rate scheduling and early stopping based on validation performance. Model selection within the grid was driven by validation balanced accuracy, with accuracy, AUROC, AUPRC, sensitivity for malignant cases (recall), and specificity for benign/negative cases recorded for all configurations. The best hyperparameter setting was then used to retrain the final model and to produce the test-set results reported in Section 3. Throughout the entire hyperparameter optimization procedure, the independent held-out test set was completely excluded from the 3-fold cross-validation scheme and was never used to guide model selection or configuration choices. Test-set evaluation was performed exclusively after the best configuration was identified and the final model was retrained on the full training + validation set.

## 3. Results

The final dataset comprised a total of 1120 breast cases derived from contrast-enhanced mammography examinations including our cases and the CDD-CESM Archive. Among these, 379 cases (33.8%) were histopathologically confirmed as malignant, while 741 cases (66.2%) were classified as benign or negative. The resulting class distribution reflects the expected imbalance encountered in real-world clinical screening and diagnostic settings and was explicitly addressed during model training through balanced sampling strategies and weighted loss functions.

Table 2 summarizes the results obtained when the models were trained on the original DICOM images, while Table 3 shows the performance achieved when the same architectures were trained on breast-mask images.

Among the models trained on DICOM images, ResNet18 CBAM achieves the best overall trade-off between sensitivity and specificity, with a balanced accuracy of 0.807, AUROC of 0.844 and AUPRC of 0.835. DenseNet121 also performs strongly, with the highest malignant recall (0.860) in the group, indicating excellent sensitivity to malignant lesions. EfficientB0 provides solid performance with relatively lightweight architecture, resulting in an excellent accuracy–complexity trade-off.

When trained on breast-mask images, all models exhibit a clear and systematic performance improvement. This gain is substantial (AUROC increases between +0.06 and +0.21 across models) and consistent across all metrics, including accuracy, balanced accuracy, AUROC and AUPRC. In particular, ResNet50 reaches the highest AUROC (0.931), while EfficientB0 attains the highest AUPRC (0.937). DenseNet121 consistently ranks among the top performing models on all metrics, and both ResNet18 and FCCSNet remain highly competitive.

The final evaluation of the held-out test set further demonstrates the capability of the model (Table 4).

**Figure 1:**
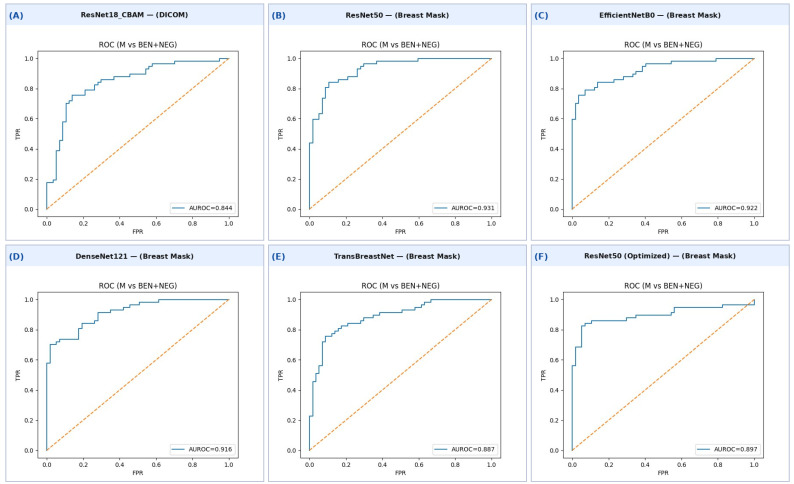
ROC curves for all evaluated models on the held-out test set (malignant vs. benign + negative). (**A**) ResNet18_CBAM (DICOM); (**B**) ResNet50 (breast mask); (**C**) EfficientNetB0 (breast mask); (**D**) DenseNet121 (breast mask); (**E**) TransBreastNet (breast mask); (**F**) ResNet50 optimized (breast mask).

The final evaluation on the held-out test set further demonstrates the capability of the model. The confusion matrix (Table 4) highlights strong performance in both malignant and benign/negative classes, achieving a balanced distribution of correct predictions. The normalized values confirm good sensitivity to malignant cases and high specificity toward non-malignant ones. The ROC curve (Figure 1A) shows an AUROC of 0.844, reflecting strong discriminative power of the model to differentiate malignant from benign/negative samples. Overall, these results validate the reliability of ResNet18_CBAM for lesion classification on unprocessed DICOM mammograms.

On the held-out test set, the ResNet50 model achieves the strongest performance among all evaluated architectures trained on breast-mask images. As reported in Table 4, the confusion matrix demonstrates high true-positive and true-negative rates, confirming robust discrimination between malignant and benign/negative samples. The ROC curve (Figure 1B) shows an AUROC of 0.931, confirming excellent discriminative power and a substantial performance gain introduced by breast-mask preprocessing.

The confusion matrix on the held-out test set (Table 4) demonstrates balanced performance across both classes, with high sensitivity for malignant lesions and strong specificity toward benign/negative cases. These results highlight the model’s ability to correctly identify malignancies while minimizing false positives. The ROC curve (Figure 1C) confirms the excellent discriminative capability of EfficientNetB0, achieving an AUROC of 0.922, the second-highest among all models trained on breast-mask images.

The confusion matrix evaluated on the held-out test set (Table 4) shows balanced performance between malignant and benign/negative classes, with 84% sensitivity for malignant lesions and 81% specificity for benign/negative cases. This balanced behavior highlights DenseNet121’s ability to correctly discriminate between the two classes while avoiding excessive false positives. The ROC curve (Figure 1D) confirms the strong discriminative capability of this architecture, with an AUROC of 0.916, comparable to that of EfficientNetB0 and only slightly lower than that of the ResNet50 variant.

The confusion matrix evaluated on the held-out test set (Table 4) shows an imbalanced error profile between malignant and benign/negative classes. In particular, the model achieves very high specificity (98%), while sensitivity for malignant lesions is markedly lower (32%). The ROC curve (Figure 1E) confirms an overall good discriminative capability, with an AUROC of 0.887. These results suggest that TransBreastNet is able to separate the classes in terms of ranking performance, but the selected operating threshold leads to reduced sensitivity to malignant lesions in the final classification. To further investigate this imbalance, additional analyses were conducted in scenarios characterized by a pronounced sensitivity–specificity asymmetry, including validation-based probability calibration and threshold adjustment strategies. Nevertheless, these approaches did not yield a substantial improvement in malignant sensitivity on the test set. This outcome indicates that the observed discrepancy between AUROC and sensitivity is not primarily attributable to suboptimal threshold selection; rather, a significant fraction of malignant cases are associated with low predicted confidence and are therefore misclassified as benign rather than lying close to the decision boundary.

Below (Table 5), we report the results of the hyperparameter optimization performed through a grid-search procedure applied to ResNet50 (the best model trained on the breast-mask dataset).

ResNet50 is a 50-layer deep convolutional neural network based on residual learning. Its key innovation is the bottleneck residual block, which enables the training of very deep architectures without suffering from vanishing gradients. Each bottleneck block contains: a 1 × 1 convolution (channel reduction), a 3 × 3 convolution (spatial feature extraction), a 1 × 1 convolution (channel expansion), and a residual skip connection added before the ReLU activation. The network contains four main stages: Conv2_x: 3 bottleneck blocks (64 channels); Conv3_x: 4 blocks (128 channels); Conv4_x: 6 blocks (256 channels); and Conv5_x: 3 blocks (512 channels). The architecture concludes with global average pooling and a fully connected classifier.

The best-performing configuration of ResNet50 was selected for retraining and final evaluation on the independent test set. The model demonstrates strong classification performance with high specificity and recall balance. The classifier correctly identifies 93% of benign and negative samples and 84% of malignant lesions. The precision–recall confirms the robustness of the classifier in high-precision regions, achieving an AUPRC of 0.929, while the ROC curve (Figure 1F) illustrates a stable trade-off between sensitivity and specificity, with an AUROC of 0.897.

In an additional exploratory analysis, the best-performing model from the binary classification task (ResNet50) was further evaluated for its ability to discriminate between Luminal A and Luminal B breast cancer subtypes, restricted to HER2-negative cases. Among malignant cases, 214 had available histological subtype information; of these, 89 cases were Luminal A or Luminal B subtypes restricted to HER2-negative status. This experiment was conducted using the same training and validation strategy adopted for the primary task. However, the model demonstrated poor diagnostic performance: confusion matrices showed inconsistent class separation across validation folds and the test set, and discriminative ability was low, with AUROC and AUPRC values close to those expected under random classification. Given these limited results and the reduced sample size of the subtype-specific cohort, no further optimization or investigation of this task was pursued.

## 4. Discussion

The present study provides robust evidence that deep learning models trained on breast-mask images derived through LIBRA segmentation significantly outperform models trained on raw DICOM mammograms. This improvement is consistent across all evaluated architectures and performance metrics, including accuracy, balanced accuracy, AUROC, AUPRC, sensitivity, and specificity. Such systematic enhancement strongly supports the hypothesis that anatomically constrained preprocessing increases the signal-to-noise ratio, reduces background variability, and highlights subtle malignant patterns that are often overshadowed in full-field mammography. These findings are in line with previous research demonstrating that segmentation-guided mammographic analysis and parenchymal isolation improve downstream classification performance by minimizing non-diagnostic structures and acquisition-related variability [7,8,48,49,50].

A key contribution of this work lies in the breadth of the architectural comparison. We evaluated eleven deep learning architectures, spanning classical CNNs, attention-enhanced models, multi-scale fusion networks, densely connected architectures, lightweight mobile models, hybrid CNN–Transformer backbones, and mammography-specific networks such as GLAM-Net and FCCSNet [43,44,45,46]. This comprehensive evaluation allowed us to assess the influence of architectural design on model performance in CEM-based lesion classification. Although specialized architectures have been increasingly proposed to address mammographic challenges [43,44,45,46], our results demonstrate that traditional CNNs remain highly competitive when the input is well preprocessed and the training pipeline is carefully optimized.

These findings further contextualize and extend our group’s prior work along two complementary lines of research. The first is our radiomic analysis [30], which demonstrated that handcrafted texture features from CEM and DCE-MRI—when combined with ensemble machine learning algorithms such as Gradient Boosting Machine—achieved an AUC of 0.907 for malignancy classification in a multicenter cohort of 153 patients. That study established the diagnostic potential of engineered imaging biomarkers extracted via the PyRadiomics platform, encompassing first-order statistics, shape descriptors, and wavelet-filtered texture matrices (GLCM, GLRLM, GLSZM). However, it was necessarily limited by sample size, dependence on manual segmentation and expert-driven feature engineering, the focus on a bimodal protocol (CEM + DCE-MRI) not universally available in clinical practice, and the absence of a systematic evaluation of end-to-end representation learning. The second is an initial engineering-oriented deep learning investigation [31], which focused exclusively on VGG16 and MobileNetV2 trained on the publicly available CDD-CESM dataset (n = 1003), demonstrating through a controlled experimental design that LIBRA-based anatomical breast masking consistently improves AUROC, sensitivity, and training stability for both high-capacity and lightweight CNN architectures. That study provided the first formal, architecture-agnostic evidence that input representation—rather than model complexity—is a primary determinant of learning behavior in CEM. However, it remained limited to two architectures, a single public dataset, and a binary classification task without multicenter validation. The present work extends both prior contributions across several complementary dimensions: (i) the transition from handcrafted radiomics to end-to-end deep feature extraction, enabling task-relevant representation learning directly from pixel-level data; (ii) a substantially larger and multicenter dataset (n = 1120), combining institutional data with the public CDD-CESM archive; (iii) the systematic evaluation of eleven deep learning architectures under rigorously controlled training conditions, spanning classical CNNs, attention-enhanced models, hybrid CNN–Transformer frameworks, and mammography-specific networks; and (iv) extended hyperparameter optimization to further characterize the interplay between preprocessing strategy, architectural choice, and training configuration. Collectively, these contributions establish the present study as an innovative and methodologically comprehensive evolution of both preceding lines of research, moving toward clinically scalable, architecturally robust, and externally validated deep learning solutions for CEM-based breast lesion characterization.

When trained on original DICOM images, models such as ResNet18 CBAM and DenseNet121 achieved strong but heterogeneous performance profiles. However, once LIBRA-generated breast-mask inputs were adopted, every architecture exhibited a marked performance gain, with AUROC increases ranging from +0.06 to +0.21. Among these models, ResNet50 achieved the highest AUROC, while EfficientNetB0 reached the highest AUPRC. DenseNet121 and MobileNetV2 also demonstrated stable and competitive results. These findings indicate that input representation may exert an influence on performance that rivals, or even exceeds, architectural sophistication.

The improvement observed with breast-mask inputs can be interpreted from a biological and imaging standpoint. CEM highlights regions of contrast uptake associated with tumor-related angiogenesis. When background tissue, pectoral muscle, and acquisition artifacts are removed, the model is constrained to focus on functional enhancement patterns and surrounding parenchymal texture. This reduces the risk of learning spurious associations, a well-recognized limitation in mammography AI systems [7,8,48,49,50], and allows even lightweight network architectures to perform at a high level.

The hyperparameter optimization stage further underscores the importance of training strategy. Differential learning rates, dropout tuning, undersampling ratio selection, and augmentation intensity all significantly influenced final performance. The optimized ResNet50 configuration achieved balanced sensitivity and specificity on the independent test set, demonstrating that well-engineered optimization procedures can elevate classical CNNs to near state-of-the-art levels, rivaling more complex hybrid architectures.

The behavior of TransBreastNet provides additional insight. Although it achieved strong threshold-independent discrimination, its operating point on the test set revealed a conservative error profile characterized by very high specificity and low malignant sensitivity. Calibration and threshold adjustment strategies did not substantially mitigate this imbalance, suggesting that the issue was not purely related to threshold selection but to intrinsic representational characteristics. Hybrid CNN-Transformer architectures [37] may require larger datasets or richer supervisory signals to fully exploit global attention mechanisms. In anatomically constrained settings such as breast-mask preprocessing, convolutional backbones may capture local enhancement patterns more effectively, while global attention modules provide limited additional benefit.

From a clinical perspective, achieving a balanced sensitivity–specificity profile is essential. High sensitivity minimizes missed cancers, whereas excessive false positives increase biopsy rates, patient anxiety, and healthcare burden. The balanced performance observed in the optimized ResNet50 model contrasts with several mammography AI studies reporting extreme operating behaviors [34], and suggests that background suppression and tissue-focused analysis promote clinically meaningful discrimination.

The performance of our optimized models is consistent with broader trends in AI-based breast imaging. Recent systems increasingly integrate attention mechanisms, hybrid CNN, Transformer backbones, and multi-view modeling strategies [37,38,39,43,44,45,46]. Additionally, generative approaches such as CycleGAN and Pix2Pix have been explored for virtual contrast enhancement in CEM [35]. Although generative pipelines were not incorporated in the present study, combining segmentation-driven preprocessing with virtual enhancement techniques represents a promising avenue for future research.

Despite these promising findings, several limitations must be acknowledged. Although multicentric and augmented with CDD-CESM public data [34], the dataset size remains moderate. The classification task was binary, whereas clinical workflows require multi-class stratification and integration of molecular or prognostic markers. Moreover, no clinical metadata or temporal imaging information were incorporated, despite evidence that multimodal integration enhances predictive modeling [37].

Overall, our findings support a representation-driven paradigm in medical AI development. In contrast-enhanced mammography, systematic engineering of input representation and rigorous optimization appear to be at least as impactful as architectural complexity in achieving clinically robust performance.

## 5. Conclusions

In conclusion, this study demonstrates that anatomically constrained preprocessing through breast-mask segmentation substantially improves deep learning performance in contrast-enhanced mammography, consistently enhancing discrimination between malignant and benign lesions. By isolating diagnostically relevant breast tissue and suppressing background variability, this approach increases robustness and clinical reliability across architectures. Moreover, our findings show that classical CNN models remain highly competitive when combined with anatomically focused inputs and rigorous optimization, often matching or outperforming more complex hybrid CNN–Transformer architectures. This suggests that data representation quality and training strategy are at least as critical as model complexity. Future work should explore the integration of breast-mask preprocessing with hybrid architectures, multimodal data fusion, temporal modeling, and generative virtual enhancement techniques to further advance AI-driven breast cancer characterization in CEM.

## Figures and Tables

**Table 1:** Overview of the trained deep learning models and their main characteristics.

Model	Architecture Type	Key Characteristics	Reference
DenseNet121	CNN (Dense)	Dense connectivity with feature reuse and reduced parameter count; enhances feature propagation and reduces the number of parameters through dense layer connectivity.	[33,41]
ResNet18	CNN (Residual)	Lightweight residual architecture with stable convergence due to skip-connections.	[36]
ResNet50	CNN (Residual)	A deep residual network known for stable training of very deep models via identity skip connections.	[33,40]
EfficientNetB0	Scaled CNN	Compound scaling strategy balancing depth, width and resolution.	[39,42]
FCCSNet	FCN + Attention	Cross-stage attention and fully convolutional design tailored for mammography.	[33,43]
VGG16	CNN (Very Deep)	Sequential 3 × 3 convolutions, high capacity, strong baseline performance.	[32,38,40]
MobileNetV2	Lightweight CNN	Depthwise separable convolutions and inverted residuals for efficient inference.	[36]
ResNet18-CBAM	CNN + Attention	ResNet18 enhanced with Channel & Spatial Attention (CBAM).	[36]
GLAMNet	CNN + Global–Local Attention	Fuses CC/MLO views using global–local attention for robust lesion localization and classification.	[33,45]
TransBreastNet	CNN + Transformer	Multi-view fusion and global context modeling.	[36,44]
ViT-Mammo	Vision Transformer	Patch-level self-attention adapted to high-resolution mammograms.	[33,46]

**Table 2:** Performance of CNN models trained on original DICOM images.

Model	BalAcc	AUROC	AUPRC	Sensitivity/Recall	Specificity
DenseNet121	0.80	0.85	0.83	0.86	0.74
TransBreastNet	0.75	0.81	0.80	0.83	0.67
FCCSNet	0.72	0.76	0.78	0.68	0.75
GLAMNet	0.75	0.82	0.80	0.77	0.74
ResNet18_CBAM	0.81	0.84	0.84	0.75	0.86
ResNet50	0.64	0.72	0.67	0.54	0.74
ResNet18	0.69	0.76	0.76	0.70	0.68
MobileNetV2	0.67	0.75	0.73	0.58	0.75
VGG16	0.72	0.83	0.80	0.61	0.83
EfficientB0	0.78	0.83	0.78	0.79	0.77
Vit_mammo	0.65	0.72	0.73	0.49	0.81

**Table 3:** Performance of CNN models trained on breast-mask images.

Model	BalAcc	AUROC	AUPRC	Sensitivity/Recall	Specificity
DenseNet121	0.83	0.92	0.93	0.84	0.81
TransBreastNet	0.83	0.87	0.88	0.79	0.86
FCCSNet	0.82	0.89	0.90	0.89	0.74
GLAMNet	0.82	0.90	0.893	0.75	0.88
ResNet18_CBAM	0.77	0.90	0.92	0.88	0.67
ResNet50	0.834	0.93	0.93	0.93	0.74
ResNet18	0.73	0.82	0.83	0.81	0.65
MobileNetV2	0.74	0.80	0.79	0.67	0.81
VGG16	0.80	0.893	0.91	0.75	0.84
EfficientB0	0.82	0.92	0.94	0.84	0.79
ViT-Mammo	0.84	0.89	0.89	0.86	0.83

**Table 4:** Confusion matrix results for all evaluated models on the independent held-out test set (57 malignant, 57 benign/negative cases). TP = true positive (correctly identified malignant); TN = true negative (correctly identified benign/negative); FP = false positive; FN = false negative. Sensitivity = TP/57; specificity = TN/57.

Model	Input	Predicted: Malignant (M)		Predicted: Benign/Neg (B/N)		Sensitivity	Specificity	AUROC
		True M	True B/N	True M	True B/N			
ResNet18_CBAM	DICOM	TP = 43	FP = 8	FN = 14	TN = 49	0.75	0.86	0.84
ResNet50	Breast Mask	TP = 53	FP = 15	FN = 4	TN = 42	0.93	0.74	0.93
EfficientNetB0	Breast Mask	TP = 48	FP = 12	FN = 9	TN = 45	0.84	0.79	0.92
DenseNet121	Breast Mask	TP = 48	FP = 11	FN = 9	TN = 46	0.84	0.81	0.92
TransBreastNet	Breast Mask	TP = 18	FP = 1	FN = 39	TN = 56	0.32	0.98	0.87
ResNet50	Breast Mask	TP = 48	FP = 4	FN = 9	TN = 53	0.84	0.93	0.90

Note: The best-performing model overall is ResNet50 (Breast Mask) for sensitivity (AUROC = 0.93), and ResNet50 Optimized for combined sensitivity (0.84) and specificity (0.93).

**Table 5:** Top 10 hyperparameter configurations from the grid search on ResNet50.

H LR	B LR	Drop	R	Aug	BalAcc	AUC	AUPRC	Recall	Spec	Youden	Thr
1 × 10^−3^	1 × 10^−4^	0.5	2	H	0.89	0.90	0.93	0.84	0.93	0.77	0.51
1 × 10^−4^	1 × 10^−4^	0.2	1.5	L	0.85	0.91	0.94	0.75	0.95	0.70	0.57
1 × 10^−4^	1 × 10^−4^	0.5	2	L	0.83	0.90	0.93	0.82	0.84	0.67	0.45
1 × 10^−3^	1 × 10^−4^	0.3	1.5	H	0.83	0.91	0.91	0.84	0.82	0.67	0.12
1 × 10^−3^	3 × 10^−5^	0.5	1.5	L	0.83	0.92	0.94	0.84	0.82	0.67	0.46
1 × 10^−3^	1 × 10^−4^	0.5	1.5	L	0.82	0.90	0.90	0.70	0.95	0.65	0.42
1 × 10^−4^	3 × 10^−5^	0.3	1.5	L	0.82	0.89	0.92	0.75	0.90	0.65	0.51
1 × 10^−4^	1 × 10^−4^	0.3	1.5	L	0.82	0.95	0.95	0.93	0.70	0.63	0.36
1 × 10^−3^	1 × 10^−4^	0.3	1.5	L	0.82	0.90	0.91	0.81	0.82	0.63	0.56
1 × 10^−4^	1 × 10^−4^	0.2	2	L	0.81	0.88	0.89	0.74	0.88	0.61	0.33

## Data Availability

The optimized ResNet50 model weights (best_ResNet50_binary.pth) and the preprocessing reference CDF map (global_cdf_map.npy) required for inference are deposited at Zenodo (https://zenodo.org/records/19589397, accessed on 23 March 2026). The CDD-CESM public dataset used in this study is available through the Cancer Imaging Archive at https://www.cancerimagingarchive.net/collection/cdd-cesm/ (accessed on 23 March 2026).
